# Alpha1-chimaerin, a Rac1 GTPase-activating protein, is expressed at reduced mRNA levels in the brain of Alzheimer's disease patients

**DOI:** 10.1016/j.neulet.2015.02.013

**Published:** 2015-02-09

**Authors:** Tomoko Kato, Yoshihiro Konishi, Shun Shimohama, Thomas G. Beach, Hiroyasu Akatsu, Ikuo Tooyama

**Affiliations:** a Molecular Neuroscience Research Center, Shiga University of Medical Science, Otsu, 520-2192, Japan; b Department of Clinical Research, Nishi-tottori National Hospital, Tottori, 689-0202, Japan; c Department of Neurology, Sapporo Medical University, Sapporo, 060-8556, Japan; d Banner Sun Health Research Institute, Sun City, Arizona, 85372, USA; e Department of Community-Based Medical Education, Nagoya City University Graduate School of Medical Sciences, Nagoya, 467-8601, Japan

**Keywords:** Alzheimer's disease, *α*1-chimaerin, Rac1, In situ hybridization, Real-time PCR

## Abstract

Alpha1-chimaerin is a GTPase-activating protein (GAP) for Rac1, a member of the Rho small GTPase family, whose action leads to the inactivation of Rac1. Rac1 activity is upregulated in Alzheimer's disease, but little is known about the role of *α*1-chimaerin. In this study, we investigated the expression and localization of *α*1-chimaerin mRNA in postmortem human brains from patients with Alzheimer's disease and control subjects. *In situ* hybridization studies demonstrated that *α*1-chimaerin was expressed by neurons in the neo-cortex of the temporal lobe and the hippocampus of both controls and Alzheimer's disease cases, with the signal intensity dramatically decreased in patients with Alzheimer's disease. Real-time PCR analysis confirmed a significant reduction of *α*1-chimaerin mRNA expression in the temporal cortex of Alzheimer's disease cases. In contrast, *α*2-chimaerin mRNA levels showed no significant difference between the groups. The present study showed reduced *α*1-chimaerin expression in the brain of Alzheimer's disease cases, suggesting a role in the upregulation of Rac1 activity during the disease process.

## 1. Introduction

Alpha1-chimaerin is a GTPase-activating protein (GAP) for Ras-related C3 botulinum toxin substrate 1 (Rac1), a member of the Rho small GTPase family [1,2]. Alpha1-chimaerin is selectively expressed in the brain, where its Rac1 GAP activity mediates Rac1 inactivation. Rac1 regulates actin polymerization, actin reorganization, cell migration, and cell cycle progression [3]. In neurons, Rac1 mediates dendritic spine formation and other morphological changes [4,5]. By inactivating Rac1, *α*1-chimaerin plays a significant role in the regulation of dendritic growth during neuronal development in the brain [6].

Alpha-chimaerin consists of two splice variants (*α*1 and *α*2). The *α*1 variant lacks the *N*-terminal SH2 domains and is more abundant in the adult brain than during development, whereas the *α*2 variant is mainly expressed in the developing brain and testes [7,8]. Increased *α*1-chimaerin promotes the pruning of dendritic branches and arbors, and *α*1-chimaerin overexpression causes loss of spines in the mouse brain [6]. In cultured hippocampus, *α*1-chimaerin inhibits the formation of new spines and removes existing spines [9]. Conversely, the down-regulation of *α*1-chimaerin increases protrusive activity from the dendrite, resulting in an increased abundance of neurons with morphologically atypical spines [6]. Therefore, *α*1-chimaerin is thought to be a regulator of dendritic spine growth, branching, and morphology that exerts its function by increasing synaptic activity via muscarinic acetylcholine receptors [6] and *N*-methyl-d-aspartate (NMDA) receptors [9], leading to pruning of dendritic arbors for precisely selected synaptic formation [6].

Alzheimer's disease (AD) is the most common cause of dementia associated with the accumulation of *ß*-amyloid (A*ß*) plaques [10,11,12,13], formation of neurofibrillary tangles [14], and neuronal death. In addition, synaptic loss is implicated as a major structural cause of cognitive dysfunction in AD [15], probably involving loss or alterations of dendritic spine formation [16]. A*ß* oligomers disrupt synaptic plasticity *in vivo* [17] and cause synaptic dysfunction in an animal model of AD [18]. A previous study has reported that Rac1 activity is upregulated in the hippocampus of AD patients [19]. However, little information is available about the expression of *α*1-chimaerin in the brain of AD patients. In this study, we therefore investigated the expression and localization of *α*1-chimaerin in postmortem brains of patients with AD and of age-matched, neuropathologically normal controls at the mRNA level.

## 2. Materials and methods

### 2.1. Subjects

All subjects were from the Banner Sun Health Research Institute Brain and Body Donation Program [20]. The tissue was processed following previously described methods [21,22]. The clinicopathological data are summarized in Table 1.

For quantitative real-time PCR, total RNA was extracted from the temporal cortex of seven sporadic AD cases (mean age ± SD, 86.3 ± 4.8 years) and eight control subjects without neurological disease (mean age ± SD, 81.3 ± 4.8 years). The mean postmortem delays for the AD cases and control subjects were 2.67 h and 2.51 h, respectively.

For in situ hybridization histochemistry, we examined the hippocampus and the temporal cortex of three sporadic AD cases (mean age ± SD, 71.7 ± 10.1 years) and three control subjects (mean age ± SD, 83.0 ± 4.4 years).

### 2.2. In situ hybridization

The hybridization probe was designed to detect the *N*-terminal region specific to *α*1-chimaerin (bases 1–175 from the initiation codon; accession number S75654). The *N*-terminal regions of *α*1-chimaerin were subcloned into the pGEM-T Easy vector (Promega, Madison, WI, USA), and the digoxigenin-UTP-labeled sense and antisense riboprobes were synthesized, according to the manufacturer's protocol.

Frozen, fixed 40 μm sections of the postmortem brai n were mounted on RNase-free silane-coated glass slides (Dako Japan Co. Ltd., Tokyo, Japan) and air-dried before immersion in diethyl pyrocarbonate-treated phosphate-buffered saline (0.1 M, pH 7.4) for 10 min. The sections were then treated for 10 min at room temperature with proteinase K (5 μg/ml) in 10 mM Tris-HCl buffer (pH 8.0) contain ing 150 mM NaCl at 37 °C, and then post-fixed with 4% paraformaldehyde in 0.1 M phosphate buffer (pH 7.4) at room temperature for 10 min. The sections were pre-hybridized for 2 h at 37 °C in a hybridization buffer [50% formamide, 5 × Denhardt's solution, 3 × saline/sodium citrate (SSC), 0.5 mg/ml yeast tRNA, and 0.5 mg/ml heat-denatured salmon sperm DNA]. The probes were diluted in the hybridization buffer to a final concentration of 2 μg/ml, and hybridization was performed for 16 h at 60 °C. After hybridization, the sections were washed for 2 h in 0.2 × SSC buffer at 60 °C, followed by rinsing in 0.1 M Tris-HCl (pH 7.5) containing 150 mM NaCl (NT buffer) for 5 min at room temperature. The sections were blocked in 1% skim milk in NT buffer for 60 min, and incubated overnight at 4 °C with alkaline phosphatase-labeled anti-digoxigenin antibody (1:200; Roche Diagnostics, Basel, Switzerland). The signal was detected using the substrates nitroblue tetrazolium chloride and 5-bromo-4-chloro-3-indolyl phosphate *p*-toluidine salt.

### 2.3. Quantitative real-time PCR

Five micrograms of each total RNA sample was reverse-transcribed for first-strand cDNA synthesis using 80 units of SuperScript II (Gibco BRL, Gaithersburg, MD, USA) and 500 pmol of oligo dT_12–18_ (Amersham Biosciences Corp., Piscataway, NJ, USA).

The reaction mixture consisted of 1 × LC-FastStart mixture, 4 mM MgCl_2_, 0.5 μM of primer, and 200 ng of cDNA. We designed a set of primers to amplify *α*1-chimaerin mRNA, but not *α*2-chimaerin, and confirmed on agarose gels that the PCR product was a single band. Real-time PCR primers for the reactions were as follows: *α*1-chimaerin, 5′-AAAATGCCATCCAAAGAGTCT-3′ (sense, 2170–2190 in GenBank accession number S75654) and 5′-GAAATTGTGAATCTTTTCATATTT-3′ (antisense, 2398– 2421 in S75654); *α*2-chimaerin, 5′-GGCTCTACTACGATGGCAAGC-3′ (sense, 297– 317 in Z22641) and 5′-CTGTAGAATCTCTCTCATCATGT-3′ (antisense, 511–533 in Z22641); ß-actin, 5′-TGGTGGGCATGGGTCAGAAGGATTC-3′ (sense, 172–196 in X00351) and 5′-CATGGCTGGGGTGTTGAAGGTCTCA-3′ (antisense, 413–437 in X00351); and MAP2, 5′-CTGTAGCAGTCCTGAAAGGTGA-3′ (sense, 454–475 in BC027583) and 5′-TGCTAGGGCAGGCTGAGCTGTATC-3′ (antisense, 718–741 in BC027583). Cycling conditions comprised an initial 10 min of incubation at 95 °C followed by 1–40 cycles of denaturation for 15 s at 95 °C, annealing for 8 s at 54 °C for *α*1-chimaerin, for 5 s at 56 °C for *α* 2-chimaerin, or for 5 s at 58 °C for ß-actin and MAP2, and extension for 15 s at 72 °C. Standard curv es were obtained from plasmids containing *α*1-chimaerin or *α*2-chimaerin cDNA. According to the Fit Points method of LightCycler, we calculated mRNA content using the standard curves. The expression levels of *α*1- and *α*2-chimaerin mRNAs in neurons were normalized to ß-actin and MAP2, respectively. Statistical analysis was performed using Student's unpaired *t* test.

## 3. Results

### 3.1. *α*1-chimaerin mRNA was downregulated in the temporal cortex of AD patients, as demonstrated by in situ hybridization

**Fig. 1:**
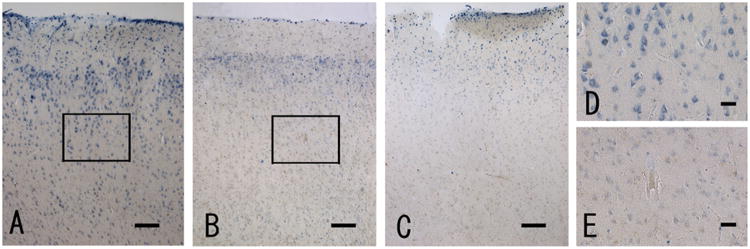
In situ hybridization of *α*1-chimaerin mRNA in the temporal cortex of control subjects (A, C and D) and AD cases (B and E) using an antisense probe (A, B, D and E) or a sense probe (C). (A) and (B): positive signals were detected in the cortex of both control subjects and AD cases. (C): no signals were detected using the sense probe with the exception of nuclear staining in areas near the cortical surface of layer 1. (D) and (E): high magnification of the boxed area in layer 3 of a control (A) and an AD case (B). Positive cells appear to be neurons. Scale bar = 200 μm in A–C, and 50 μm in D and E.

In situ hybridization detected *α*1-chimaerin mRNA in the temporal cortex of both control subjects (Fig. 1A) and AD cases (Fig. 1B). No signal was detected using the sense probe with the exception of nuclear staining in areas near the cortical surface of layer 1 (Fig. 1C). At high magnification, positive signals were localized to neurons (Fig. 1D and 1E, supplementary Fig. S1). Although there was no difference in the localization of *α*1-chimaerin mRNA between control subjects and AD cases, the signal intensity dramatically decreased in the cerebral cortex of AD patients (Fig. 1B and 1E), compared with those of control subjects (Fig. 1A and 1D).

**Fig. 2:**
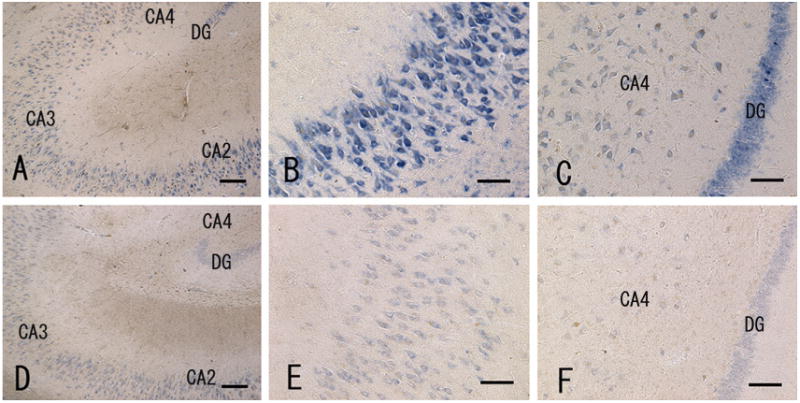
In situ hybridization of *α*1-chimaerin mRNA in the hippocampus of a control subject (A–C) and AD case (D–F). (A) and (D): at low magnification, positive signals were mainly visible in the pyramidal layers of the cornu ammonis (CA) and granular cell layer of the dentate gyrus (DG) in both control subjects and AD cases. (B) and (E): high magnification of the pyramidal layer of the CA2 region. (C) and (F): high magnification of the granular cell layer of the dentate gyrus. Signal intensity is reduced in the AD case (D–F) relative to that in controls (A–C). Scale bar = 200 μm in (A) and (D); 100 μm in (B, C, E and F).

In the hippocampus of control cases (Fig. 2), *α*1-chimaerin mRNA was strongly expressed in the pyramidal neurons of the cornu ammonis (CA) (Fig. 2A and 2B) and in the granule cells of the dentate gyrus (DG) (Fig. 2C); however, this signal intensity was dramatically decreased in pyramidal neurons (Fig. 2D and 2E) and granule cells (Fig. 2F) of AD cases.

### 3.2. Reduced *α*1-chimaerin mRNA levels in the temporal cortex of AD patients were confirmed by real-time PCR analysis

**Fig. 3:**
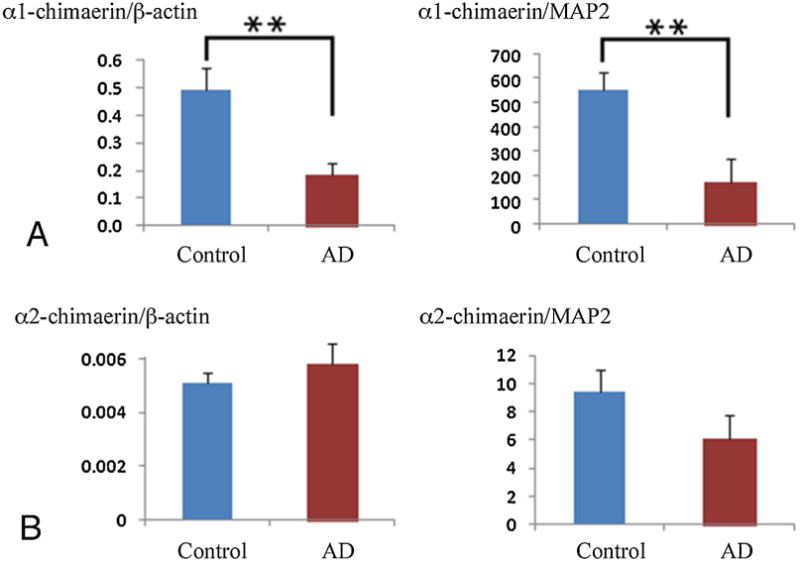
mRNA expression levels of *α*1-chimaerin (A) and *α*2-chimaerin (B) in the temporal cortex of patients with AD (*n* = 7) and control cases (*n* = 8) using real-time PCR. The mRNA expression levels of *α*1-chimaerin and *α*2-chimaerin are normalized to the ß-actin and MAP2 mRNA levels. The mRNA expression level of *α*1-chimaerin, but not *α*2-chimaerin, is significantly reduced in the temporal cortex of AD cases compared to controls. Results are presented as mean ± SEM. Statistical analysis was performed using Student's *t*-test: **P < 0.01 vs. control cases.

Real-time PCR was used to compare the expression levels of *α*1-chimaerin mRNA between AD cases and control subjects (Fig. 3). Statistical data are presented in Table 2. We used 200 ng of cDNA from the temporal cortex for the real-time PCR, and calculated the contents according to a standard curve generated using the *α*1-chimaerin plasmid. The expression levels of *α*1-chimaerin mRNA in control subjects and AD cases were 1185.49 ± 163.97 fg (mean ± SEM, *n* = 8) and 263.75 ± 134.12 fg (mean ± SEM, *n* = 7), respectively. When normalized to ß-actin and MAP2, *α*1-chimaerin mRNA levels in the AD cases were reduced to 37.3% and 31.3%, respectively, compared to control levels (*P < 0.01*, Table 2 and Fig. 3A).

We also examined the expression of *α*2-chimaerin mRNA using real-time PCR. The relative expression levels of *α*2-chimaerin mRNA in the control subjects and AD cases were 18.53 ± 1.22 (mean ± SEM, *n* = 8) and 8.95 ± 2. 22 (mean ± SEM, *n* = 7), respectively, with no significant difference detected between the groups after normalization to ß-actin and MAP2 (Table 2 and Fig. 3B).

## 4. Discussion

This study is the first to demonstrate the localization of *α*1-chimaerin mRNA in the brains of human AD patients and control subjects, and to compare the expression levels of *α*1-chimaerin mRNA between temporal cortex samples from both groups. In situ hybridization histochemistry demonstrated *α*1-chimaerin mRNA in the temporal cortex neurons of both control subjects and AD cases, with the AD brains showing a reduced signal intensity of *α*1-chimaerin mRNA compared to controls. The neuronal localization of *α*1-chimaerin is consistent with previous reports using rats [2,7,8]. In the rat brain, *α*1-chimaerin mRNA is expressed specifically in neurons and expression rapidly increases postnatally [8], although interestingly, expression was high in the cortex, including the entorhinal cortex and hippocampus, and the amygdala [8]. These regions are known to be vulnerable in AD.

In agreement with the in situ hybridization results, the real-time PCR analysis confirmed a significantly reduced expression level of *α*1-chimaerin mRNA in the temporal cortex of AD brains compared to controls. When the expression level was normalized to that of MAP2 mRNA, *α*1-chimaerin mRNA expression was significantly reduced in the AD brains. These results suggest that the reduction of *α*1-chimaerin is not due simply to neuronal loss, but that it could reflect a pathological mechanism.

When normalized to ß-actin and MAP2, the expression of *α*2-chimaerin mRNA showed no significant difference between the AD cases and control subjects. These results are in good agreement with a previous study using the rat brain [8], and suggest that *α*1-chimaerin is the main form of this GAP protein in the adult brain.

The precise roles of *α*1-chimaerin in the pathology of AD are not revealed in this study. However, there are several possibilities. Synapse development and plasticity are controlled by Rho GTPase regulatory proteins [23], and chimaerins are one class of Rho GAPs with a GAP domain specific for Rac. GAPs are generally thought to downregulate the activity of small GTPases such as Rac1, as active GTP-bound forms become inactive GDP-bound forms. Therefore, a reduction in *α*1-chimaerin would be expected to increase Rac1 activity at that location. This is in agreement with a previous paper showing that Rac1 activity was increased in the brain of AD patients [19]. In addition, increased expression of Rac1b, a constitutively active splice variant of Rac1, increased only within neurons in AD [24]. Taking together, these findings suggest that the alterations in *α*1-chimaerin and Rac1 in AD brains could be one of the mechanisms underlying synaptic dysfunction.

The other possibility is that *α*1-chimaerin is associated in some way with cyclin-dependent kinase 5 (Cdk5). Cdk5 is a neuron-specific Rac effector [25], and *α*1-chimaerin exists in a functional complex with Cdk5 in the brain [26]. Cdk5 is thought to be involved in the phosphorylation and aggregation of tau protein, tangle formation, and A*ß* neurotoxicity in the brain of AD patients [27,28]. Thus, it will be of great interest to clarify the interaction between *α*1-chimaerin and Cdk5.

## 5. Conclusion

In this study, we investigated the expression and localization of *α*1-chimaerin mRNA in postmortem brains from patients with AD and control subjects. In situ hybridization studies demonstrated that *α*1-chimaerin was expressed by neurons in the temporal lobe and the hippocampus, and staining intensity was reduced in AD cases. Real-time PCR analysis confirmed a significant reduction of *α*1-chimaerin mRNA expression in the brain of AD cases compared to controls, while there was no significant difference in *α*2-chimaerin mRNA levels between the groups.

## Supplementary Material

1

## Figures and Tables

**Table 1:** Clinicopathological data of study subjects.

Case #	Pathological diagnosis	Age (years)	Gender	Postmortem delay (*h*)	Clinical diagnosis and complications	CERAD neuritic plaque score	Braak neurofibrillary tangle stage	Analysis
1	Control	81	F	3.0	Myocardial infarction, congestive heart failure, renal failure	A	II	qPCR
2	Control	78	M	2.7	Coronary artery disease, chronic, obstructive pulmonary disease, congestive heart failure, diabetes mellitus, hip bone fracture, cardiac pacemaker	0	II	qPCR
3	Control	82	F	2.0	Lung cancer, myocardiac infarction	A	II	qPCR
4	Control	73	F	1.5	Ovarian cancer	0	I	qPCR
5	Control	85	F	2.5	Cardiac and respiratory failure, atrial fibrillation, cerebrovascular, accident (right hemiplegia)	0	III	qPCR
6	Control	78	M	1.7	Lung cancer, heart failure	0	I	qPCR, ISH
7	Control	85	M	3.2	Congestive heart failure	0	II	qPCR, ISH
8	Control	88	F	3.0	Chronic renal failure	0	II	qPCR
9	Control	86	M	2.5	Renal failure, attrial fabrillation, coronary artery disease, congestive heart failure	0	II	ISH
10	AD	89	F	3.0	AD, osteoarthritis, anxiety, depression, aspiration pneumonia	C	V	qPCR
11	AD	79	M	2.0	AD	C	V	qPCR
12	AD	89	F	3.0	AD bronchopneumonia	C	V	qPCR
13	AD	80	F	2.2	AD, parkinsonism	C	VI	qPCR
14	AD	91	F	3.0	AD	C	V	qPCR
15	AD	87	M	3.0	AD parkinsonism	C	V	qPCR
16	AD	89	F	2.5	AD	C	VI	qPCR
17	AD	61	F	2.5	AD, uterus cancer, bronchitis	C	VI	ISH
18	AD	73	F	2.0	AD, atrial fibrillation, cerebrovascular accident	C	V	ISH
19	AD	81	M	3.0	AD, cardiac and respiratory failure,	C	V	ISH

AD: Alzheimer's disease, qPCR: quantitative polymerase chain reaction, ISH: in situ hybridization histochemistry.

**Table 2:** Alpha-chimaerin mRNA expression levels in the temporal cortex of Alzheimer's disease cases and control subjects.

	Control (*n* = 8)	Alzeimer's disease	*P*-value
*α*1-chimaerin/ß-actin	0.50 ± 0.08	0.18 ± 0.04	0.0053**
*α*1-chimaerin /MAP2	553.09 ± 70.00	173.1 ± 94.7	0.0060**
*α*2-chimaerin/ß-actin	0.0051 ± 0.0004	0.0058 ± 0.0008	0.407
*α*2-chimaerin/MAP2	9.40 ±1.56	6.13 ±1.61	0.169

Values are presented as mean ±SEM.

** *P*<0.01.

## Highlights

1. Alpha1-chimaerin mRNA was localized to neurons in postmortem human brain.
2. Reduced mRNA levels of *α*1-chiamerin in the temporal cortex and hippocampus of AD.
3. Expression of *α*2-chimaerin mRNA was not reduced in the temporal cortex of AD.

## References

[1] Hall C, Monfries C, Smith P, Lim HH, Kozma R, Ahmed S, Vanniasingham V, Leung T, Lim L (1990). Novel human brain cDNA encoding a 34,000 Mr protein n-chimaerin, related to both the regulatory domain of protein kinase C and BCR, the product of the breakpoint cluster region gene. J Mol Biol.

[2] Lim HH, Michael GJ, Smith P, Lim L, Hall C (1992). Developmental regulation and neuronal expression of the mRNA of rat n-chimaerin, a p21rac GAP:cDNA sequence. Biochem J.

[3] Bosco EE, Mulloy JC, Zheng Y (2009). Rac1 GTPase: a “Rac” of all trades. Cell Mol Life Sci.

[4] Luo L, Hensch TK, Ackeman L, Barbel S, Jan LY, Jan YN (1996). Differential effects of the Rac GTPase on Purkine cell axons and dendritic trunks and spines. Nature.

[5] Nakayama AY, Harms MB, Luo L (2000). Small GTPases Rac and Rho in the maintenance of dendritic spines and branches in hippocampal pyramidal neurons. J Neurosci.

[6] Buttery P, Beg AA, Chih B, Mason CA, Scheiffele P (2006). The diacylglycerol-binding protein *α*1-chimaerin regulates dendritic morphology. Proc Natl Acad Sci USA.

[7] Hall C, Sin WC, Teo M, Michael GJ, Smith P, Dong JM, Lim HH, Manser E, Spurr NK, Jones TA, Lim L (1993). *α*2-chimerin, an SH2-containing GTPase-activating protein for the ras-related protein p21rac derivated by alternate splicing of the human *n*-chimerin gene, is selectively expressed in brain and testes. Mol Cell Biol.

[8] Hall C, Michael GJ, Cann N, Ferrari G, Teo M, Jacobs T, Monfries C, Lim L (2001). Alpha2-chimaerin, a cdc42/rac1 regulator, is selectively expressed in the rat embryonic nervous system and is involved in neuritogenesis in N1E-115 neuroblastoma cells. J Neurosci.

[9] Van de Ven TJ, VanDongen HMA, VanDongen AMJ (2005). The nonkinase phorbol ester receptor alpha1-chimerin binds the NMDA receptor NR2A subunit and regulates dendritic spine density. J Neurosci.

[10] Selkoe DJ (1991). The molecular pathology of Alzheimer's disease. Neuron.

[11] Hardy JA, Higgins GA (1992). Alzheimer's disease: the amyloid cascade hypothesis. Science.

[12] Citron M, Oltersdorf T, Haass C, McConlogue L, Hung AY, Seubert P, Selkoe DJ (1992). Mutation of the beta-amyloid precursor protein in familial Alzheimer's disease increases beta-protein production. Nature.

[13] Hardy J, Selkoe DJ (2002). The amyloid hypothesis of Alzheimer's disease: progress and problems on the road to therapeutics. Science.

[14] Bancher C, Brunner C, Lassmann H, Budka H, Jellinger K, Wiche G, Seitelberger F, Grundke-Iqbal I, Wisniewski HM (1989). Accumulation of abnormally phosphorylated τ precedes the formation of neurofibrillary tangles in Alzheimer's disease. Brain Res.

[15] Selkoe DJ (2002). Alzheimer's disease is a synaptic failure. Science.

[16] Knobloch M, Mansuy IM (2008). Dendritic spine loss and synaptic alterations in Alzheimer's disease. Mol Neurobiol.

[17] Walsh DM, Kiyubin I, Fadeeva JV, Cullen WK, Anwyl R, Wolfe MS, Rowan MJ (2002). Naturally secreted oligomers of amyloid *ß* protein potently inhibit hippocampal long-term potentiation in vivo. Nature.

[18] Perez-Cruz C, Nolte MW, van Gaalen MM, Rustay NR, Termont A, Tanghe A, Kirchhoff F, Ebert U (2011). Reduced spine density in specific regions of CA1 pyramidal neurons in two transgenic mouse models of Alzheimer's disease. J Neurosci.

[19] Zhu X, Raina AK, Boux H, Simmons ZL, Takeda A, Smith MA (2000). Activation of oncogenic pathways in degenerating neurons in Alzheimer's disease.

[20] Beach TG, Sue LI, Walker DG, Roher AE, Lue L, Vedders L, Connor DJ, Sabbagh MN, Rogers J (2008). The Sun Health Research Institute Brain Donation Program: description and experience, 1987–2007. Cell Tissue Bank.

[21] Beach TG, Sue LI, Scott S, Sparks DL (1998). Neurofibrillary tangles are constant in aging human nucleus basalis. Alzheimer's Reports.

[22] Tooyama I, Sato H, Yasuhara O, Kimura H, Konishi Y, Shen Y, Walker DG, Beach TG, Sue LI, Rogers J (2001). Correlation of the expression level of C1q mRNA and the number of C1q-positive plaques in the Alzheimer disease temporal cortex: analysis of C1q mRNA and its protein using adjacent or nearby sections. Dement Geriatr Cogn Disord.

[23] Tolias KF, Dumen JG, Um K (2011). Control of synapse development and plasaticity by Rho GTPase regulatory proteins. Prog Neurobiol.

[24] Perez SE, Getova DP, He B, Counts SE, Geula C, Desire L, Coutadeur S, Peillon H, Ginsberg SD, Mufson EJ (2012). Rac1b increases with progressive tau pathology within cholinergic nucleus basalis neurons in Alzheimer's Disease. Am J Pathol.

[25] Nikolic M, Chou MM, Lu W, Mayer BJ, Tsai LH (1998). The p35/Cdk5 kinase is a neuron-specific Rac effector that inhibits Pak1 activity. Nature.

[26] Qi RZ, Ching YP, Kung HF, Wang JH (2004). *α*-Chimaerin exists in a functional complex with the Cdk5 kinase in brain. FEBS Lett.

[27] Cruz JC, Tsai LH (2004). A Jekyll and Hyde kinase: roles for Cdk5 in brain development and disease. Curr Opin Neurobiol.

[28] Tsai LH, Lee MS, Cruz J (2004). Cdk5, a therapeutic target for Alzheimer's disease?. Biochem Biophys Acta.

