# Supplementary material for: Alpha1-chimaerin, a Rac1 GTPase-activating protein, is expressed at reduced mRNA levels in the brain of Alzheimer's disease patients
Source: Neurosci Lett. Author manuscript; Available in PMC 2016 Mar 30. (PMC4382517; doi:10.1016/j.neulet.2015.02.013)
Supplement: 1 [file NIHMS662691-supplement-1.docx]

Supplementary Data

**Double staining for α1-chimaerin and HuC/HuD**

Some tissue sections were also double stained for α1-chimaerin by ISH and for HuC/HuD by immunohistochemistry. The entire coding region of α1-chimaerin was subcloned into the pGEM-T Easy vector (Promega, Madison, WI, USA), and after linearization with *Nco*I and *Sal*I, the respective digoxigenin-UTP labeled sense and antisense riboprobes were transcribed using the SP6 and T7 RNA polymerases according to the manufacturer’s protocol. The riboprobes were subsequently purified by ethanol precipitation and digested to approximately 400 bp by alkaline lysis.

*In situ* hybridization was employed as described in the manuscript. After hybridization, sections were incubated simultaneously with alkaline phosphatase-labeled goat anti-digoxigenin antibody (1:200; Roche Diagnostics, Basel, Switzerland) and mouse anti-HuC/HuD antibody (1:500; Molecular Probes, Eugene, OR) diluted in NT buffer containing 1% skim milk at 4˚C. After washing with TBST, the sections were incubated for 4 h with Alexa Fluor 568-conjugated chicken anti-mouse IgG (1:500; Molecular Probes) at room temperature. After washing, the mounted sections were analyzed by laser scanning confocal microscopy using a Zeiss LSM 510 (Carl Zeiss, Thornwood, NY). The sections were then rinsed in NT buffer and visualized after reaction with the substrates NBT and BCIP.


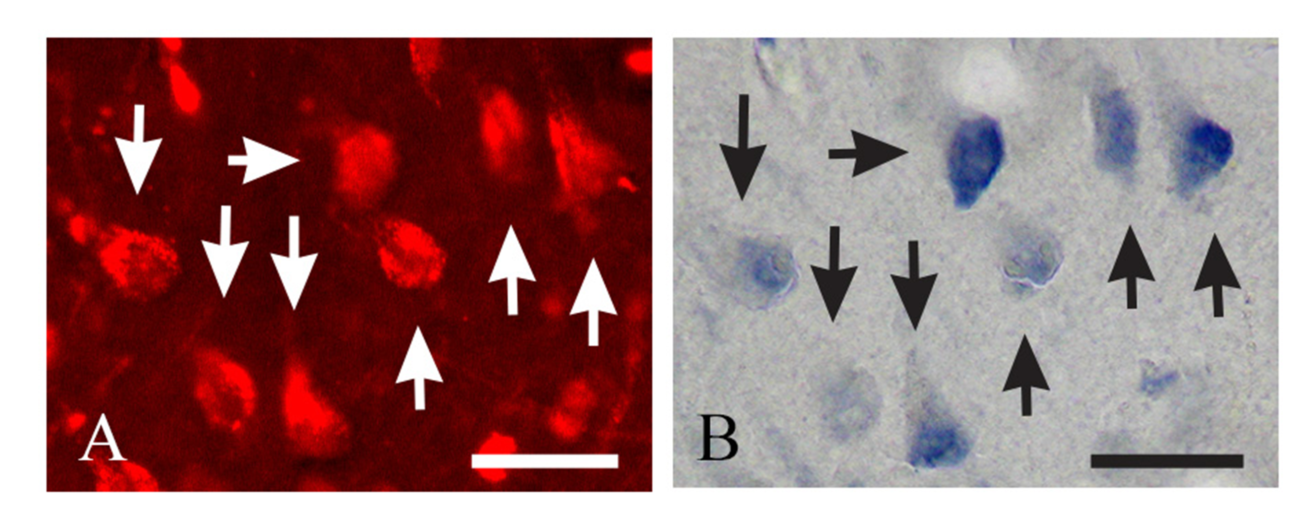


Fig. S1. Double staining for HuC/HuD by immunofluorescence (A) and α1-chimaerin (B) by in situ hybridization in the temporal lobe of a control case. Alpha 1-chimaerin signals are visible in HuC/HuD-labeled neurons (arrows). Bar = 50 µm.
